# Highly Active Microbial Phosphoantigen Induces Rapid yet Sustained MEK/Erk- and PI-3K/Akt-Mediated Signal Transduction in Anti-Tumor Human γδ T-Cells

**DOI:** 10.1371/journal.pone.0005657

**Published:** 2009-05-21

**Authors:** Daniel V. Correia, Francisco d'Orey, Bruno A. Cardoso, Telma Lança, Ana R. Grosso, Ana deBarros, Leila R. Martins, João T. Barata, Bruno Silva-Santos

**Affiliations:** 1 Molecular Immunology Unit, Instituto de Medicina Molecular, Faculdade de Medicina da Universidade de Lisboa, Lisbon, Portugal; 2 Cancer Biology Unit, Instituto de Medicina Molecular, Faculdade de Medicina da Universidade de Lisboa, Lisbon, Portugal; 3 Cellular Biology Unit, Instituto de Medicina Molecular, Faculdade de Medicina da Universidade de Lisboa, Lisbon, Portugal; 4 Instituto Gulbenkian de Ciência, Oeiras, Portugal; New York University School of Medicine, United States of America

## Abstract

**Background:**

The unique responsiveness of Vγ9Vδ2 T-cells, the major γδ subset of human peripheral blood, to non-peptidic prenyl pyrophosphate antigens constitutes the basis of current γδ T-cell-based cancer immunotherapy strategies. However, the molecular mechanisms responsible for phosphoantigen-mediated activation of human γδ T-cells remain unclear. In particular, previous reports have described a very slow kinetics of activation of T-cell receptor (TCR)-associated signal transduction pathways by isopentenyl pyrophosphate and bromohydrin pyrophosphate, seemingly incompatible with direct binding of these antigens to the Vγ9Vδ2 TCR. Here we have studied the most potent natural phosphoantigen yet identified, (E)-4-hydroxy-3-methyl-but-2-enyl pyrophosphate (HMB-PP), produced by *Eubacteria* and *Protozoa*, and examined its γδ T-cell activation and anti-tumor properties.

**Methodology/Principal Findings:**

We have performed a comparative study between HMB-PP and the anti-CD3ε monoclonal antibody OKT3, used as a reference inducer of *bona fide* TCR signaling, and followed multiple cellular and molecular γδ T-cell activation events. We show that HMB-PP activates MEK/Erk and PI-3K/Akt pathways as rapidly as OKT3, and induces an almost identical transcriptional profile in Vγ9^+^ T-cells. Moreover, MEK/Erk and PI-3K/Akt activities are indispensable for the cellular effects of HMB-PP, including γδ T-cell activation, proliferation and anti-tumor cytotoxicity, which are also abolished upon antibody blockade of the Vγ9^+^ TCR Surprisingly, HMB-PP treatment does not induce down-modulation of surface TCR levels, and thereby sustains γδ T-cell activation upon re-stimulation. This ultimately translates in potent human γδ T-cell anti-tumor function both *in vitro* and *in vivo* upon transplantation of human leukemia cells into lymphopenic mice,

**Conclusions/Significance:**

The development of efficient cancer immunotherapy strategies critically depends on our capacity to maximize anti-tumor effector T-cell responses. By characterizing the intracellular mechanisms of HMB-PP-mediated activation of the highly cytotoxic Vγ9^+^ T-cell subset, our data strongly support the usage of this microbial antigen in novel cancer clinical trials.

## Introduction

The capacity to recognize and eliminate transformed cells is common to several lymphocyte subsets of both the adaptive and the innate immune systems that are being targeted in cancer immunotherapy [1], [2]. One population that appears to bridge these two systems in humans is characterized by the expression of a Vγ9Vδ2 T-cell receptor and represents 1–10% of peripheral blood lymphocytes (PBL) of healthy individuals, but expands up to 30–50% upon bacterial or protozoan infection [3].

In line with the cancer susceptibility phenotype of mice devoid of γδ T-cells [4], human Vγ9Vδ2 T-cells are endowed with notable anti-tumor activity toward a large spectrum of malignant cell lines of diverse tissue origin, particularly among lymphomas and leukemias [5], but also including melanomas and carcinomas [6], and are being explored in various clinical trials [7], [8]. Unexpectedly, Vγ9Vδ2 cells were shown to respond to self- and foreign *non*-peptidic low molecular weight antigens with phosphate moieties (“phosphoantigens”), in what turns out to be an exclusive property of this lymphocyte subset [9], [10], [11]. Indeed, no other human T-cell subset (namely Vδ1 cells), or any of the murine γδ populations, respond to phosphoantigens such as prenyl pyrophosphates [3].

From its early isolation from mycobacteria, isopentenyl pyrophosphate (IPP) [10] became the model phosphoantigen for studies on Vγ9Vδ2 activation. However, it is now clear that this class of compounds contains multiple members, either naturally occurring or synthetic, which span an extremely diverse range of bioactivities, up to 10^10^ fold differences. To date, the natural phosphoantigen with highest bioactivity known (32 picomolar) is (E)-4-hydroxy-3-methyl-but-2-enyl pyrophosphate (HMB-PP), an intermediate of the 2-C-methyl-D-erythritol 4-phosphate (MEP) pathway employed by *Eubacteria* and apicomplexan *Protozoa* but not by eukaryotes [12]. Although HMB-PP is respectively 30,000 and 100 times more potent than IPP and bromohydrin pyrophosphate (BrH-PP, also known as “Phosphostim”), most of the studies on phosphoantigens have been performed with these compounds (already applied in the clinic) due to their historical precedence [3]. Such studies revealed a very slow kinetics of activation of TCR-associated signal transduction pathways, and conflicting results regarding their potential interactions with the Vγ9Vδ2 TCR [12], [13], [14]. This, added to the consistent failure to demonstrate cognate interactions between Vγ9Vδ2 TCRs and phosphoantigens in acellular systems [15], has shed some skepticism regarding the action of phosphoantigens as direct TCRγδ agonists. As HMB-PP is considered for γδ T-cell-based cancer clinical trials, hoping to improve the performance of previous phosphoantigens [7], [8], it is crucial to clarify its own molecular/cellular mechanisms of action, including its potential capacity to trigger *bona fide* Vγ9Vδ2 TCR signaling. Consistent with such potential, it has been recently shown that HMB-PP has the capacity to induce the formation of high-density TCR nanoclusters on the surface of human γδ T-cells [16], and a newly-developed tetramer reagent for the Vγ9Vδ2 TCR of rhesus macaques was reported to bind to HMB-PP loaded on the surface of human antigen presenting cells (APC) [17].

In this study we have analyzed the intracellular effects of HMB-PP stimulation of human γδ T-cells. Our data show that HMB-PP induces the activation of MEK/Erk and PI-3K/Akt signaling pathways with similar kinetics to direct cross-linking of the TCR complex in human γδ T-cells, and requires those activities to mediate effective γδ T-cell activation, including a full repertoire of TCR-associated transcriptional signatures and the secretion of pro-inflammatory cytokines IFN-γ and TNF-α. Although TCR accessibility is required for HMB-PP activity, this phosphoantigen does not lead to ligand-induced TCR internalization, which appears to be advantageous for sustaining the cells' activation status upon re-stimulation. Finally, very low amounts of HMB-PP in conjugation with interleukin-2 (IL-2) confers human γδ T-cells with very potent anti-lymphoma/leukemia activity both *in vitro* and in a human/SCID mouse model for the transplantation of human tumors, thus attesting the therapeutic potential of HMB-PP for cancer immunotherapy.

## Results

### Nanomolar amounts of HMB-PP replicate saturating TCR/CD3 ligation for activation of Vγ9^+^ T-cells

**Figure 1:**
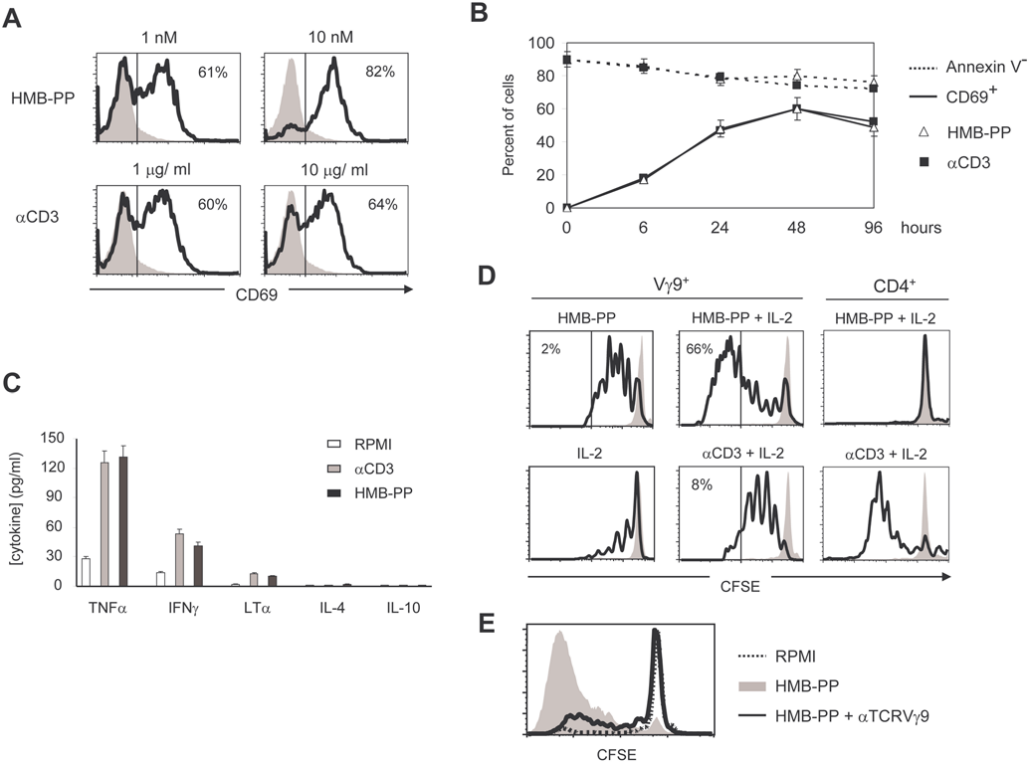
Nanomolar HMB-PP replicates saturating TCR/CD3 ligation for Vγ9^+^ T-cell activation. (A) Flow cytometry analysis for the expression of the activation marker CD69 in MACS-sorted (97–98% purity) γδ PBL, stimulated for 48 hours with the indicated amounts of HMB-PP or anti-CD3 mAb (OKT3). Shaded are non-stimulated Vγ9^+^ T-cells. Percentages refer to cells above the threshold bar. (B) Time-course of the experiment described in (A) for 1 nM HMB-PP and 1 µg/ml OKT3; cells were also stained with Annexin V to assess their viability (Annexin V^−^). (C) Cytokine bead array analysis of supernatants of MACS-sorted γδ PBL (of which 80–90% Vγ9^+^) cultures after 24 hours of stimulation with HMB-PP or OKT3. RPMI refers to cells kept in media not supplemented with activating compounds. (D) CFSE dilution assays to monitor T-cell proliferation in total PBMC cultures supplemented with HMB-PP (1 nM) or OKT3 (1 µg/ml), with or without 100 U/mL rhIL-2. Cells (gated on Vγ9^+^ or CD4^+^) were analyzed by flow cytometry after 4 days in culture; shaded are non-divided cells. Percentages indicate cells that have undergone more than 5 rounds of division. (E) CFSE dilution in gated Vγ9^+^ T-cells within 6-day cultures of total PBMC activated with 1 nM HMB-PP in the presence or absence of blocking anti-TcRVγ9 antibody. Dashed is a control incubated in 10% RPMI without HMB-PP. Results shown in this figure are representative of 3 independent experiments.

In this study we used the anti-CD3ε monoclonal antibody (αCD3 mAb) OKT3 as a control for canonical T-cell activation through the TCR/CD3 complex, for direct comparison with HMB-PP. We began by testing the effect of several doses of each stimulating compound on human γδ T-cell activation, proliferation and survival. Concentrations of 1 nM HMB-PP and 1 µg/ml OKT3 produced identical profiles of expression of the activation marker CD69 in the Vγ9^+^ T-cell population (Figure 1A), and displayed strikingly similar kinetics of activation without significant differences in cell viability (Figure 1B); they were therefore used in all subsequent experiments. Interestingly, whereas αCD3 mAb treatment reached a plateau of 60% CD69^+^ cells at 1–10 µg/ml OKT3, 10 nM of HMB-PP were able to further increase the abundance of activated Vγ9^+^ T-cells, to above 80% (Figure 1A).

Activated Vγ9Vδ2 T-cells are known to secrete large amounts of IFNγ and TNFα, very potent anti-tumor mediators *in vivo*. In accordance, treatment of sorted γδ PBL (80–95% Vγ9^+^) with HMB-PP induced a typical Th1 cytokine profile, characterized by the preferential production of TNFα, IFNγ and LTα, in the absence of significant IL-4 or IL-10 (Figure 1C). Notably, the levels of Th1 cytokines produced after 1 nM HMB-PP treatment were similar to those induced by saturating amounts of αCD3 mAb (Figure 1C and data not shown), suggesting that low amounts of this phosphoantigen are able to fully exploit the TCR-mediated functional potential of Vγ9Vδ2 T-cells.

For the selective expansion of Vγ9^+^ T-cells, HMB-PP has the advantage of not inducing αβ T-cell proliferation. Thus, HMB-PP treatment promoted the specific proliferation of Vγ9^+^ T-cells within human PBL (Figure 1D). Importantly, this effect was completely abolished upon addition of a blocking antibody to the Vγ9^+^ TCR (Figure 1E), demonstrating the TCR-dependence of HMB-PP activity.

While HMB-PP alone promoted up to 5 divisions of Vγ9^+^ T-cells over 4 days, further proliferation required the co-administration of IL-2 (Figure 1D). A cooperative effect between phosphoantigens and IL-2 has been previously described [18], [19], and in this study translated into a Vγ9^+^ T-cell expansion of 30-fold within one week and 45-fold within two weeks of stimulation (Figure S1A). Moreover, addition of 100 units/mL IL-2 to HMB-PP cultures dramatically increased the total amounts of Th1 cytokines secreted by γδ T-cells by 20–80 fold (Figure S1B), which correlated with the induction of key transcription factor *t-bet* in cells stimulated with IL-2 or IL-2/HMB-PP combination (Figure S1C)

### HMB-PP rapidly triggers MEK/Erk and PI-3K/Akt signaling required for Vγ9^+^ T-cell activation and anti-tumor function

**Figure 2:**
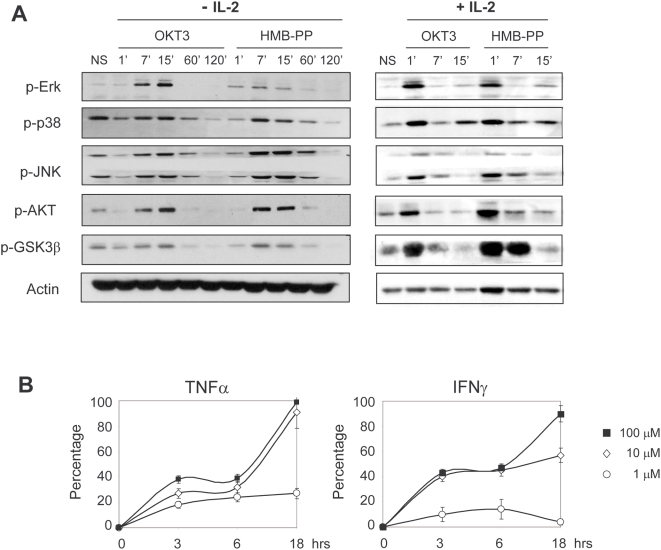
HMB-PP stimulation kinetically mimics Vγ9^+^ TCR/CD3 signal transduction. (A) Phosphoimmunoblotting for kinases implicated in TCR signaling. MACS-sorted γδ PBL (of which 80–95% Vγ9^+^) were incubated with OKT3 (1 µg/ml) or HMB-PP (1 nM), in the absence (*left panel*) or presence (*right panel*) of 100 U/mL rhIL-2, for the times indicated, or kept in control media (NS, non-stimulated). Results shown in this figure are representative of 4 independent experiments. (B) TNFα and IFNγ levels were measured by CBA in the culture supernatants of MACS-sorted γδ PBL. Results were compared with the total amounts present in parallel cultures stimulated with 1 nM HMB-PP, and were expressed as percentages (IPP/HMB-PP).

Having characterized the cellular behavior of HMB-PP-stimulated Vγ9^+^ T-cells, we next investigated the intracellular signaling mechanisms downstream of HMB-PP. Previous studies with less active phosphoantigens [3] reported a significant delay in the activation of kinase cascades when compared to direct TCR/CD3 complex ligation with OKT3 mAb [12], [13]. Instead, for HMB-PP, we observed a very rapid (peaking around 7 min of stimulation), and absolutely identical to OKT3, kinetics of phosphorylation of the major signaling pathways implicated in TCR signal transduction: JNK, Erk and p38 MAPK; and PI-3K-associated Akt and GSK3β (Figure 2A, left panel). The same was valid in the presence of IL-2, in which kinase phosphorylation peaked earlier (immediately after 1 min of stimulation) but was still identical for HMB-PP or OKT3 combinations (Figure 2A, right panel). Of note, we verified that IPP could not replicate these signaling properties of HMB-PP, as illustrated by its failure to induce Akt phosphorylation within 60 minutes of stimulation (Figure S2). Furthermore, IPP treatment (even when used at 10^5^ fold higher concentrations than HMB-PP) resulted in a modest production of TNFα and IFNγ within the first 6 hours of stimulation, when compared to HMB-PP (Figure 2B). These data reveal a thus far unique capacity of HMB-PP to trigger very rapid TCR-associated signaling, compatible with direct binding of the phosphoantigen to the TCR complex.

**Figure 3:**
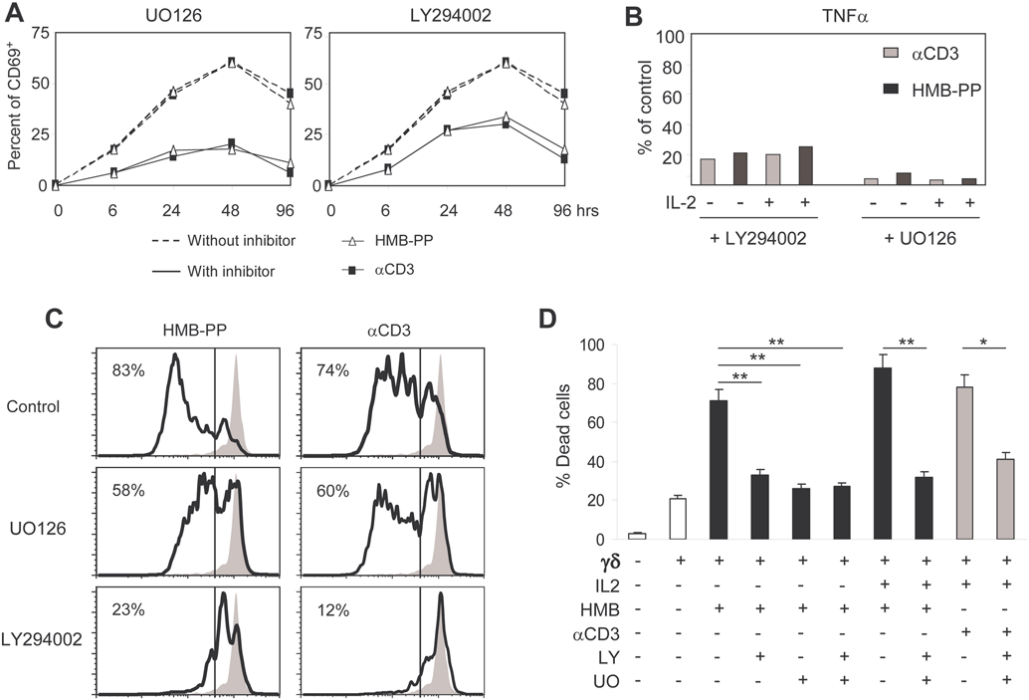
HMB-PP-mediated Vγ9^+^ T-cell activation requires functional MEK/Erk and PI-3K/Akt signaling pathways. Effects of MEK/Erk inhibitor UO126 and PI-3K/Akt inhibitor LY294002 on the activation and function of MACS-sorted γδ PBL (of which 85–95% Vγ9^+^). (A) Expression of activation marker CD69, assessed by flow cytometry. (B) secretion of TNFα after 24 hours of stimulation, measured by CBA. (C) Cell proliferation, assessed by CFSE dilution after 4 days in culture (percentages indicate cells that have undergone 2 or more rounds of division). (D) Jurkat leukemia cell killing, assessed by Annexin V staining and flow cytometry analysis after 6 hrs of co-incubation with pre-activated (for 3 days) γδ PBL. Results shown in this figure are representative of 3 independent experiments. Error bars represent SD and significant differences refer to controls without addition of chemical inhibitors (n = 3, *p<0.05 and **p<0.01).

We next tested the requirement on intact PI-3K and MAPK pathways for γδ T-cell activation and anti-tumor function induced by HMB-PP. We pre-treated γδ T-cells with chemical inhibitors that specifically block those pathways and then analyzed the effects on cell activation, proliferation, TNFα secretion and tumor cell killing. Inhibition of PI-3K/Akt pathway using LY294002 resulted in approximately half of the cells losing their responsiveness to HMB-PP after 24–46 hours of stimulation (Figure 3A). Inhibition of the MEK/Erk pathway by UO126 produced even more dramatic effects, precluding HMB-PP-activation of approximately two thirds of Vγ9^+^ T-cells. Moreover, inhibition of PI-3K/Akt and MEK/Erk signaling reduced TNFα production by HMB-PP-activated γδ T-cells to around 20% and 10% of control levels, respectively, both in the absence and in the presence of IL-2 (Figure 3B). These effects were remarkably mirrored in cultures supplied with αCD3 mAb, further demonstrating the similarity of these two activation regimens (Figures 3A and 3B).

In what regards γδ T-cell proliferation induced either by HMB-PP or by OKT3 (in the presence of IL-2), this was mostly dependent on intact PI-3K/Akt signaling, since UO126 had a more modest effect when compared with the severe block produced by LY294002 treatment, which reduced the proportion of γδ cells that divided twice or more over 4 days in culture, from over 80% to approximately 20% (Figure 3C).

Finally, the anti-tumor function of sorted γδ PBL (80–95% Vγ9^+^) was assessed through *in vitro* killing of the Jurkat leukemic target cell line. HMB-PP pre-treatment augmented γδ T-cell-mediated tumor cell death from around 20% (non-activated γδ) to 40% (1 nM HMB-PP) or 70% (10 nM HMB-PP) in a 6 hour assay (Figure 3D and data not shown). However, the addition of UO126 or/and LY294002 to the treatment reduced posterior leukemia targeting to basal (20–30%) levels; this was also the case for the more efficient (over 80% killing) combination of HMB-PP with IL-2 (Figure 3D). Collectively, these data demonstrate an absolute requirement of PI-3K/Akt- and MEK/Erk-mediated signal transduction for HMB-PP-induced activation of anti-tumor Vγ9Vδ2 T-cells.

### HMB-PP signaling mimics the transcriptional events downstream of TCR ligation

**Figure 4:**
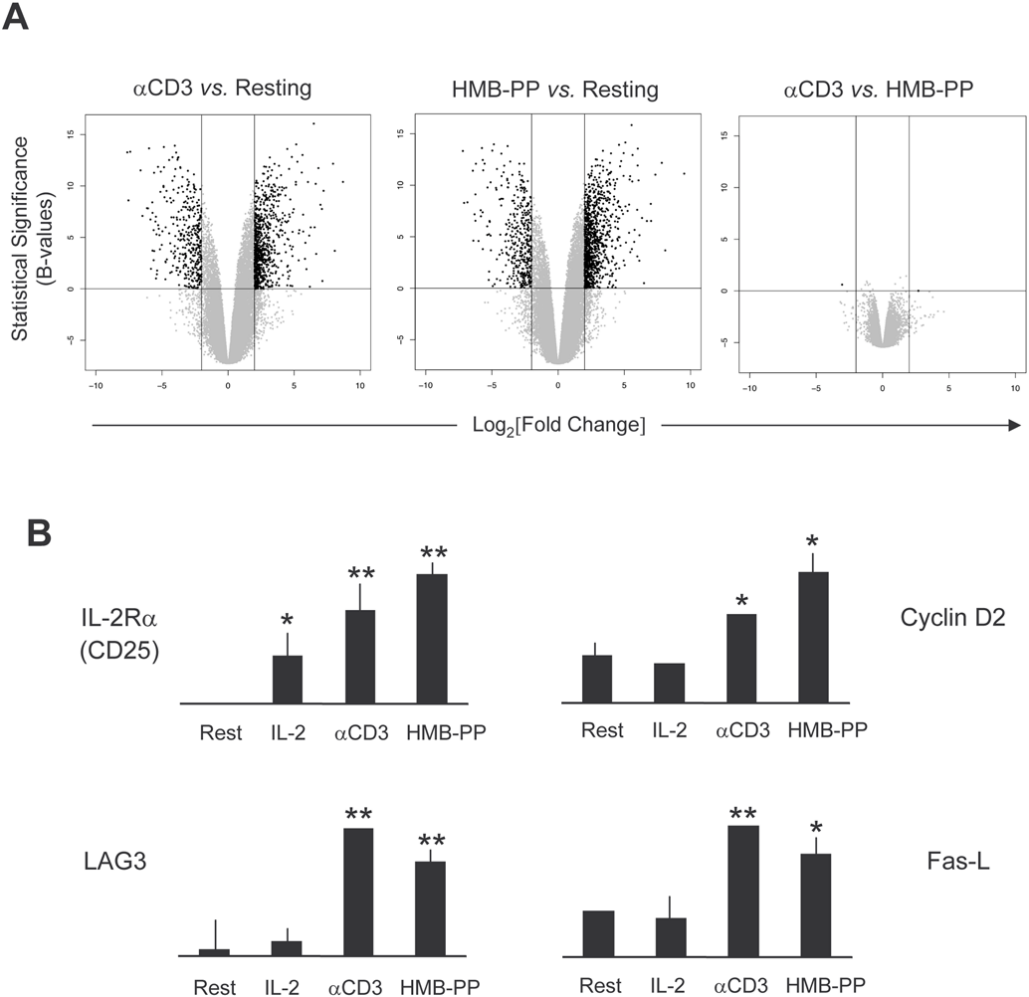
HMB-PP treatment reproduces the transcriptional alterations induced by TCR/CD3 ligation on γδ T-cells. (A) Volcano plots of DNA microarray comparisons between αCD3 (OKT3) mAb-treated, HMB-PP-treated and non-stimulated (“resting”) MACS-sorted γδ PBL (of which 85–95% Vγ9^+^). After 18 hours of incubation with the stimuli, RNA was extracted and submitted to Affymetrix GeneChip analysis. Represented are Fold-changes (“biological significance”) versus statistical significance (*B*-values). Black dots represent genes over 4-fold differentially expressed (DE) between samples; all other probed genes are depicted in grey. Genes selected as differentially expressed had adjusted *p*-values lower than 0.005. Results are representative of 3 independent microarray experiments (see Figure S2). (B) Real-time PCR validation of microarray results for a selection of genes similarly induced by OKT3 and HMB-PP (from Table 1). Gene expression was quantified in independent samples of control and treated cells, also including an IL-2-treated sample. Error bars represent SD and significant differences refer to “resting” cells (n = 3, *p<0.05 and **p<0.01).

Signaling cascades ultimately produce alterations in gene transcription, which can be effectively tracked by microarray analysis. We employed this technology to compare the transcriptomes of Vγ9Vδ2 T-cells activated with either HMB-PP or OKT3. Both stimuli produced dramatic transcriptional changes: when compared to non-stimulated cells, HMB-PP and OKT3 treatment resulted in 1359 and 1080 differences in gene expression of 4-fold or above, respectively (Figure 4A; full microarray data available on ArrayExpress via http://www.ebi.ac.uk/; accession E-MEXP-1601). These were consistent across 3 individual microarray experiments (Figure S2). Strikingly, a direct comparison of the two stimuli revealed that they affected essentially the same genes, as only 6 were differentially expressed (>4-fold) between them (Table 1). Therefore, the transcriptional program downstream HMB-PP appears to be extremely similar to that induced by *bona fide* TCR signaling, as clearly illustrated by the Volcano plots of Figure 4A.

**Table 1:** Transcriptional changes induced by HMB-PP or OKT3 (anti-CD3 mAb) in Vγ9Vδ2 T cells.

Similarly induced by HMB-PP and OKT3(a)						
Link^b^	Gene	Description	Function	HMB c	OKT3 c	Differ d
3458	*IFNγ*	Interferon-γ	Cytokine	9.53	8.70	0.83
6355	*CCL8*	Chemokine CC motif 8	Chemokine	8.09	8.08	0.01
114614	*MIRN155*	MicroRNA 155	MicroRNA	7.83	7.95	−0.12
4049	*LTα*	Lymphotoxin-α	Cytokine	7.35	6.64	0.71
6347	*CCL2*	Chemokine CC motif 2	Chemokine	6.49	6.19	0.30
3559	*IL2Rα*	IL-2R α chain	Cytokine-R	6.34	7.10	−0.76
4283	*CXCL9*	Chemokine CXC motif 9	Chemokine	6.14	6.14	0.00
3627	*CXCL10*	Chemokine CXC motif 10	Chemokine	5.75	4.87	0.88
894	*CCND2*	Cyclin D2	Cell cycle	5.62	5.25	0.37
3902	*LAG3*	Lymphocyte activation gene	Activation-R	4.46	4.08	0.38
29851	*ICOS*	Inducible T cell costimulator	Activation-R	4.46	5.40	−0.94
6504	*SLAMF1*	Signal transducer SLAM-1	Signaling	4.23	4.34	−0.11

Differentially induced by HMB-PP or OKT3						
Link^b^	Gene	Description	Function	HMB c	OKT3 c	Differ d
7412	*VCAM1*	Vascular cell adhesion-R	Adhesion	4.50	1.45	3.05
6373	*CXCL11*	Chemokine CXC motif 11	Chemokine	5.45	2.59	2.86
1493	*CTLA4*	Co-receptor CTLA-4	Activation-R	4.53	7.23	−2.70
112744	*IL17F*	IL-17 isoform F	Cytokine	1.29	4.81	−3.52
4094	*MAF*	Transcription factor Maf	Signaling	0.04	3.83	−3.79
6374	*CXCL5*	Chemokine CXC motif 5	Chemokine	0.04	7.14	−7.10

Values are log_2_[fold change] compared to non-stimulated cells, based on triplicate microarray experiments. (-R, receptor).

a Listed is a selection of genes implicated in T cell activation. Full cDNA microarray data available on ArrayExpress (E-MEXP-1601).

b Locus link gene ID (for unequivocal gene identification).

c Log_2_[fold change] relative to non-stimulated cells.

d Difference in fold induction between HMB-PP-treated and OKT3-treated cells.

The gene expression program shared by HMB-PP treatment and direct TCR cross-linking involves, among many others targets (E-MEXP-1601), the very high (above 16-fold) up-regulation of pro-inflammatory genes IFNγ and LTα, chemokines CCL8, CCL2, CXCL9 and CXCL10, cell cycle mediator cyclin D2, activation co-receptor ICOS, cytolysis mediator Fas ligand (Fas-L), and components of cytokine receptors IL-2Rα (CD25) and IL-15Rα (Table 1), many of which are also induced by related phosphoantigens [20]. These results were validated by quantitative real-time PCR (qPCR), as shown on Figure 4B for a selection of genes.

Although we have concentrated here on genes upregulated upon stimulation, the profile of downregulated genes was also almost identical between the two treatments (E-MEXP-1601). Our results collectively suggest that HMB-PP essentially recapitulates the transcriptional program associated with *bona fide* TCR signaling. This phenomenon is further illustrated by a heatmap representation of gene expression levels across the samples, as depicted in Figure S3.

### HMB-PP does not induce Vγ9^+^ TCR internalization and sustains the production of anti-tumor cytokines

**Figure 5:**
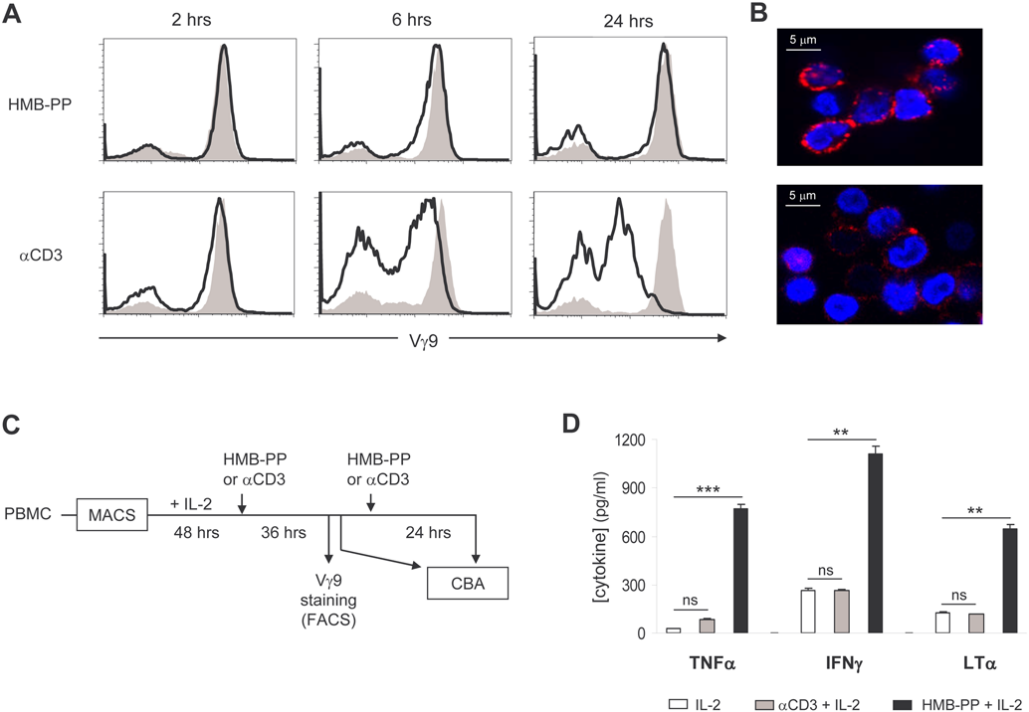
HMB-PP does not induce down-modulation of surface Vγ9^+^ TCR and sustains cytokine production upon re-stimulation. (A) MACS-sorted γδ PBL (of which 80–90% Vγ9^+^) were incubated for the indicated times with HMB-PP or OKT3, and stained with anti-Vγ9 mAb for flow cytometry analysis. Bold lines represent treated cells, while shaded are non-stimulated Vγ9^+^ cells (time = 0 hrs). (B) Confocal microscopy photos of γδ T-cells cultured for 24 hrs as in (A) and then stained for Vγ9^+^ TCR. (C–D) Experimental design (C) and CBA analysis (D) of the re-stimulation response of MACS-sorted γδ PBL. After 36 hrs of stimulation, cells were re-plated for secondary activation during 24 hrs, when supernatants were collected and analyzed for Th1 cytokines by CBA. Error bars represent SD and differences refer to IL-2 controls (ns, non-significant; **p<0.01; ***p<0.001). Results shown in this figure are representative of 2–5 independent experiments.

Although our previous data demonstrated a striking parallel between HMB-PP- and OKT3-mediated γδ T-cell activation, previous reports on various phosphoantigens (other than HMB-PP) had revealed contradictory data on the modulation of surface Vγ9Vδ2 TCR levels [13], [14]. This, added to recent data on the properties of HMB-PP interactions with TCR/CD3 complexes [16], [17], prompted our investigation on whether HMB-PP stimulation induced TCR internalization in human γδ PBLs. In αβ T cells, activation by cognate antigen or anti-TCR/CD3 antibodies typically induces TCR internalization and consequently the down-modulation of its surface levels independently of the constitutive recycling of the complex [21], [22]. Using two independent approaches, based on flow cytometry (Figure 5A) or confocal microscopy (Figure 5B), we consistently observed that HMB-PP-stimulated γδ T-cells maintained their high TCR surface expression, in stark contrast with the extensive down-regulation seen in OKT3-treated cells. This was the case both in the absence and in the presence of IL-2 (data not shown).

We next asked whether the lack of TCR internalization upon HMB-PP treatment could be associated with sustained activation of γδ T-cells. We tested the capacity of cells that had been treated for 2 days with either HMB-PP or OKT3, to respond to a second boost of stimulation (Figure 5C). Whereas HMB-PP-treated cells, which maintained high TCR levels on the cell surface after the initial 48 hour treatment (Figure 5A), produced high amounts (similar to primary activation) of anti-tumor Th1 cytokines in response to the secondary 24 hour stimulation with HMB-PP (Figure 5D), OKT3-treated cells failed to do so, presumably due to their inability to respond to the mAb once their TCR complexes have been internalized (Figure 5A–B). Although upon restimulation with HMB-PP, IFNγ became more abundant than TNFα (Figure 5D), contrary to the primary activation data (Figure 1C and Figure S1B), the cytokine profile of the two HMB-PP-based protocols were qualitatively very similar and consistently Th1-biased (Figure 5D and data not shown). These data show that HMB-PP is remarkably capable of sustaining Vγ9Vδ2 T-cell activation and the production of anti-tumor cytokines, which are critical parameters in immunotherapy protocols.

### HMB-PP plus IL-2 treatment promotes leukemia cell killing *in vitro* and *in vivo*

**Figure 6:**
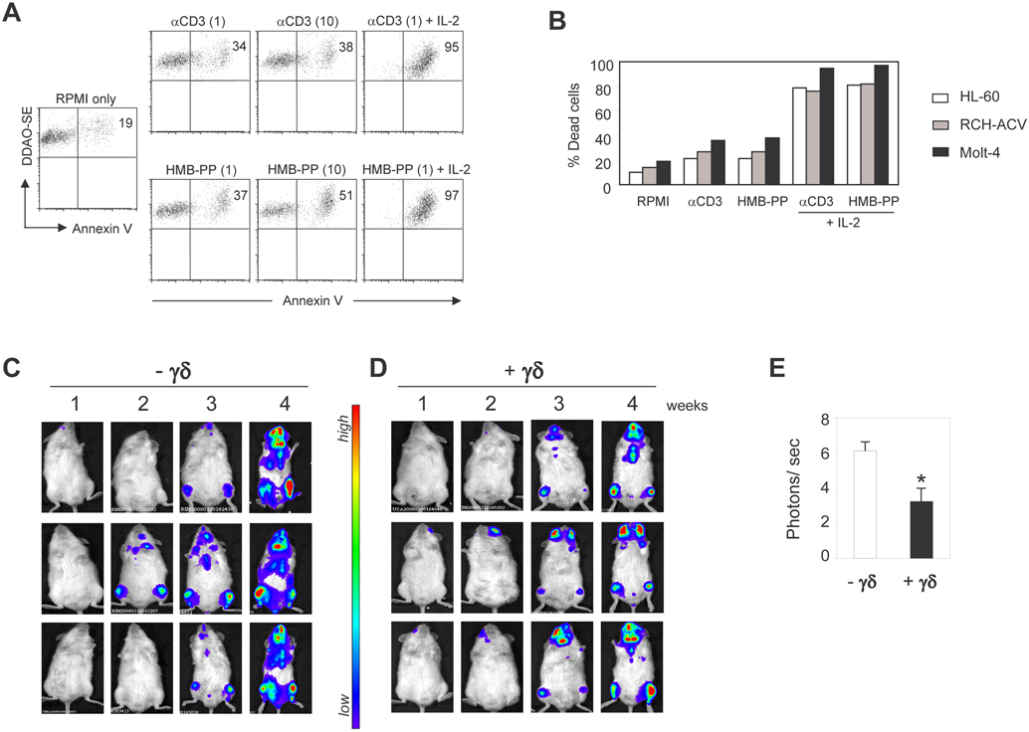
Leukemia cell killing by HMB-PP-activated γδ T-cells. (A) *In vitro* lysis of Molt-4 leukemia cells. MACS-sorted γδ PBL (of which 85–95% Vγ9^+^) were pre-activated for 72 hours with 1 or 10 µg/ml αCD3 mAb (OKT3), or 1 or 10 nM HMB-PP in the absence of IL-2, and also combined at the lower concentrations with IL-2 (100 U/ml). For the killing assay, DDAO-SE-labelled Molt-4 cells and pre-activated γδ PBL were co-incubated for 3 hours in media devoid of activating compounds. Samples were then stained with Annexin V to identify dying (Annevin V^+^) tumor (DDAO-SE^+^) cells by flow cytometry. (B) Data summary for killing assays (as in A) performed with three distinct leukemia cell lines. (C–D) Bioluminescent imaging of NOD/SCID mice inoculated with luciferase^+^ Molt-4 leukemic cells, with (D) or without (C) co-injection of pre-activated γδ PBL, analyzed weekly as described in Materials and Methods. (E) LivingImage quantification of photon signals (tumor load) collected at day 28 of the experiment illustrated in (C–D). Comparison of γδ-treated and control animals (n = 5, p<0.05). Data in this figure are representative of 3 (A–B) or 2 (C–E) independent experiments.

Having characterized the intracellular mechanisms of HMB-PP-mediated γδ T-cell activation, we next evaluated the anti-tumor potential of HMB-PP-based regimens. We selected leukemias as model tumors to employ in both *in vitro* and *in vivo* assays. The *in vitro* system previously used with Jurkat cells (Figure 3D) was applied to a larger panel of leukemia cell lines: Molt-4 (T-cell), RCH-ACV (pre-B cell) and HL-60 (myeloid) (Figures 6A–B). γδ PBL (80–95% Vγ9^+^) were treated with the different stimulating agents for 72 hours, and then transferred to plain media in co-culture with the leukemia cells. In just 3 hours, more than 80% of leukemia cells were killed by the γδ T-cells that had been stimulated with a combination of HMB-PP with IL-2 (compared to less than 20% by non-activated γδ T-cells), and such a regimen was at least as effective as saturating αCD3 plus IL-2 (Figures 6A–B). Of note, αCD3 mAb or HMB-PP used in isolation produced more modest increases in target-cell lysis (Figures 6A–B), highlighting the importance of exogenous IL-2 for the full activation of Vγ9Vδ2 T-cells [18], [23] (Figure S1).

Taking into account the added relevance of pre-clinical *in vivo* systems for the evaluation of the anti-tumor potential of immunotherapy strategies, we adapted a model of transplantation of human tumors into lymphopenic SCID mice, previously used with human γδ T-cells by Kabelitz and colleagues [24], and added bioluminescent analysis of tumor development, which allows early detection of tumors and temporal evaluation throughout the course of treatment, in live animals and in real-time [25]. Four weeks after tumor injection, mice that had received HMB-PP plus IL-2-treated (activated and expanded over 12 days) γδ PBL showed significantly reduced tumor load (derived from Molt-4 leukemia cells) compared to control mice that did not receive γδ T-cells (Figures 6C–E). Furthermore, while most control had to be sacrificed at week 4 due to excessive body weight loss, γδ-treated animals resisted wasting for longer, up to week 6 (Figures 6C–D and data not shown). These results attest the capacity of HMB-PP-expanded and activated γδ T-cells to induce anti-tumor responses *in vivo*, and support the application of this phosphoantigen in conjugation with low amounts of IL-2 in clinical cancer settings.

## Discussion

The stimulatory effect prenyl pyrophosphates have on Vγ9Vδ2 T-cells has been well documented and seems to require TCR expression, as indicated by antibody blocking and gene transfer experiments [26], [27]. However, some of these experiments have been difficult to reproduce, and all attempts at showing cognate interactions between Vγ9Vδ2 TCRs and phosphoantigens in acellular systems (including surface plasmon resonance and X-ray crystallography of isolated complexes) have failed [15], probably due to the requirement of an unknown phosphoantigen-presenting molecule [12]. This has raised some skepticism on phosphoantigens as TCRγδ agonists, also stemming from the lack of precedent for such type of compounds interacting with any other variable region molecule, including all other γδ TCRs in humans or mice. However, recent data have highlighted particular properties of HMB-PP within the large family of phophoantigens. Namely, HMB-PP induces the formation of high-density Vγ9Vδ2 TCR nanoclusters on the membrane of human γδ T-cells [16], and is bound on the surface of human APC by a tetramer reagent for the Vγ9Vδ2 TCR of rhesus macaques [17].

Following from these results on the extracellular dynamics of HMB-PP, our study aimed at clarifying the intracellular mechanisms of γδ T-cell activation mediated by HMB-PP. Our results show that very low amounts of HMB-PP are able to mimic the major effects of saturating ligation of the TCRγδ/CD3 complex, including the very rapid activation of MEK/Erk and PI-3K/Akt pathways to set up a transcriptional program, further enhanced by IL-2 signaling, that upregulates crucial target genes such as IFNγ or TNFα and endows cells with potent anti-tumor capacity. Interestingly, HMB-PP can produce all these intracellular events without down-modulating surface TCR levels, and this may be advantageous for sustaining the cells' activation status upon re-stimulation, as suggested by our cytokine secretion data. The crucial effect of HMB-PP on Vγ9Vδ2 T-cells may thus be the formation of high-density surface TCR nanoclusters [16] that may serve as platforms for intracellular signaling.

Our kinetic data on signal transduction further suggest that the interaction between HMB-PP and the Vγ9Vδ2 TCR is much more direct/stable than those of previously studied phosphoantigens, since downstream Erk phosphorylation, for example, peaks simultaneously for HMB-PP and OKT3 treatments, in stark contrast with the delays of 115 min and 60 min, also relative to OKT3, observed respectively for the “pioneer” (naturally-occurring) IPP [13] and the more recent (synthetic) BrH-PP [12], currently in clinical trials as “Phosphostim”. Of note, the concentration of HMB-PP we used was 50,000-fold and 3,000-fold *lower* than those used for IPP and BrH-PP, respectively. These data reveal a thus far unique capacity of HMB-PP to trigger very rapid TCR-associated signaling, compatible with direct binding to the Vγ9Vδ2 TCR, which remains to be formally shown and may require the assistance of an antigen-presenting molecule yet to identify [12], [17]. This notwithstanding, we show for the first time, using a chemical inhibition strategy, that the major cellular effects of HMB-PP - γδ T cell activation, proliferation, Th1 cytokine secretion and anti-tumor cytotoxicity - are strictly dependent on Erk- and Akt-mediated signal transduction. HMB-PP stimulation therefore recruits the same signal transduction machinery employed by the γδ TCR, and is capable of doing so at minimal concentrations and within a strikingly short temporal scale that distinguish it from less potent phosphoantigens, whose stimulating effects on γδ T-cells most probably derive from their structural similarities with HMB-PP [3].

The use of a microbial compound for the activation of human anti-tumor lymphocytes fits the overall strategy of providing immune adjuvants (like viral nucleic acids for CD8^+^ T-cells) for cancer therapy. Compared to other T-cell agonists, HMB-PP offers the advantage of specifically activating a T-cell population with overt effector function, devoid of known immune suppressive (“regulatory”) subsets. Moreover, Vγ9Vδ2 T-cells are broadly reactive to tumors, potentially allowing them to be used to treat a variety of cancers.

The data presented in this report provide a framework for designing novel immunotherapy protocols using γδ T-cells, and encourage the use of HMB-PP in clinical settings. γδ T-cell-mediated tumor surveillance should evidently be seen as complementary to the adaptive component provided by MHC-restricted αβ T-cells upon priming by dendritic cells. Importantly, Vγ9Vδ2 T-cells can also induce monocyte and DC maturation [28], [29], [30], on one hand; and even act as CD80/86-expressing antigen-presenting cells that prime αβ T-cells, on the other [31]. Furthermore, γδ T-cells are prototypic representatives of unconventional lymphocytes with innate anti-tumor capacity, alike NK and NKT-cells, all of which recognize tumors independently of classical MHC presentation [2]. We believe the success of cancer immunotherapy will critically depend on the integration of conventional and unconventional lymphocyte responses [32] to tackle the multiple immune evasion strategies developed by tumors.

## Materials and Methods

### Ethics statement

All experiments involving animals (rodents) were performed in compliance with the relevant laws and institutional guidelines and have been approved by the Instituto de Medicina Molecular animal ethics committee.

### *In vitro* cultures of human peripheral blood lymphocytes

Peripheral blood was collected from anonymous healthy volunteers, diluted 1∶1 (v/v) with PBS(1×) (Invitrogen Gibco) and centrifuged in LSM Lymphocyte Separation Medium (MP Biomedicals) in a volume ratio of 3∶4 (3 parts of LSM for 4 of diluted blood) for 15 minutes at 1500 rpm and 25°C. The interfase containing PBMC was collected, washed in PBS (1×) and cultured at 1×10^6^ cells/mL at 37°C, 5% CO2 in round-bottom 96 well plates with RPMI 1640 with 2 mM L-Glutamine (Invitrogen Gibco) supplemented with 10% foetal bovine serum (Invitrogen Gibco), 1 mM Sodium Pyruvate (Invitrogen Gibco), 50 mg/mL of penicillin/streptomycin (Invitrogen Gibco), in the presence or absence of 100 U/mL of rhIL-2 (Roche Applied Science), 1–10 nM of HMB-PP (4-hydroxy-3-methyl-but-2-enyl pyrophosphate) (a kind gift from H. Jomaa and M. Eberl), and 1-10 ug/ml of soluble anti-CD3 antibody (eBioscience, clone OKT3).

For TCR blockade, freshly-isolated PBMC were CFSE-labeled and then incubated for 6 days with anti- TcRVγ9 (Beckman Coulter, clone IMMU360) diluted 1∶20 in complete medium supplemented with 1 nM HMB-PP.

For the phophoimmunoblotting experiments, MACS-isolated γδ T cells were expanded with 100 U/mL rhIL-2 for 15 days.

To study the effects of chemical inhibitors of signal transduction, the MEK inhibitor UO126 and the PI-3K inhibitor LY294002 (both from Calbiochem) were added at 10 µM for a 2-hour incubation period, and then transferred to fresh medium (without inhibitors).

### Magnetic cell sorting and flow cytometry analysis

γδ T-cells were isolated (to above 95% purity) from PBMC by magnetic cell sorting via positive selection with a FITC-labeled anti-TCRγδ antibody (Miltenyi Biotec). For flow cytometry analysis (on a FACSCalibur, BD Biosciences), cells were labelled with fluorescent monoclonal antibodies: anti-CD69-PE (BD Pharmingen), anti-TcRVγ9-PC5 (Beckman Coulter) and anti-CD4-PerCP (BD Pharmingen). In all cultures the percentage of Vγ9^+^ T-cells was evaluated by flow cytometry. Cell proliferation was measured by following a standard CFSE staining protocol (CellTrace CFSE Cell Proliferation Kit, Invitrogen; final concentration 0.5 µM), while apoptosis was assessed by AnnexinV-FITC (BD Pharmingen) staining. Cells were counted in Mossbauer chambers using 0.4% Trypan Blue solution (Sigma-Aldrich) for viability control.

### Cytometric Bead Array (CBA)

Cytokine secretion was measured using Cytometric Bead Array (CBA) technology (BD Biosciences). Cells were seeded with the respective activators at 2×10^5^ cells/well, culture supernatants were collected at different time points and analyzed on a FACSCanto (BD Biosciences) using a custom-made Flex Set with five different cytokine capture beads: LT-α, IL-10, IL-4, TNF-α and IFN-γ. Data were analyzed using the FCAP Array Software v1.0.1 running on BD FACSDiva (BD Biosciences).

### Protein isolation and phosphoimmunoblotting

Cells were incubated at 37°C with pre-warmed PBS alone or with HMB-PP (1 nM) or OKT3 (1 µg/mL). Reactions were stopped by placing samples on ice and adding ice-cold PBS. Cell lysates were prepared and equal amounts of protein were analyzed by 10% SDS-PAGE electrophoresis, transferred onto nitrocellulose membranes, and immunoblotted with the following mAbs or antisera: Actin, phospho-Erk (Y204) (Santa Cruz Biotechnology), ZAP-70 and phospho-STAT5A/B (Y694/Y699) (Upstate Biotechnology), phospho-Akt (S473), phospho-GSK-3β (S9), phospho-JNK/SAPK (Y183/185), phospho-p38 MAPK (Y180/182) (Cell Signalling Technology), and phospho-LCK (Y505) (Transduction Laboratories). Immunodetection was performed with horseradish peroxidase-conjugated secondary antibody and developed by chemiluminescence as described [33]. Whenever necessary membranes were striped using 15 mM TRIS pH 6.8 plus 2% SDS and β-Mercaptoethanol (100 mM) for 40 minutes at 57°C.

### RNA isolation and Affymetrix GeneChip analysis

RNA labeling, hybridization to the Affymetrix GeneChip Human Genome U133 plus 2.0 Arrays and scanning was performed by the Affymetrix Core Facility, Instituto Gulbenkian de Ciencia, Portugal as described below.

Total RNA was extracted using the RNeasy Mini Kit according to manufacture's protocol (Qiagen, Hilden, Germany). Concentration and purity was determined by spectrophotometry and integrity was confirmed using an Agilent 2100 Bioanalyzer with a RNA 6000 Nano Assay (Agilent Technologies, Palo Alto, CA). RNA was processed for use on Affymetrix (Santa Clara, CA, USA) GeneChip Human Genome U133 Plus 2.0 Arrays, according to the manufacturer's One-Cycle Target Labeling Assay. Arrays were scanned on an Affymetrix GeneChip scanner 3000 7G.

All the microarray data analysis was done using R and several packages available from CRAN (R Development Core Team, 2008) and Bioconductor. The raw data (CEL files) was normalized and summarized with the Robust MultiArray Average method from the *affy* package.

The differentially expressed genes were selected using linear models and empirical Bayes methods as implemented in *limma* package, verifying the *p*-values corresponding to moderated F-statistics, and selecting as differentially expressed genes those that had adjusted *p*-values lower than 0.005.

### Real-time quantitative PCR

Total RNA was reverse-transcribed into cDNA using random hexamers and Superscript II first strand synthesis reagents (Invitrogen). qPCR was performed on ABI Prism 7700 Sequence Detection System using SYBR Green detection system (both from PE Applied Biosystems). Primers were designed using Primer3 v.0.4.0 online program (http://primer3.sourceforge.net). Primer sequences are available upon request. For each transcript, quantification was done using the calibration curve method. β2-microglobulin was used as the internal control for normalization. All samples were run in triplicate and repeated three times. Analysis of the qPCR results was performed using the ABI SDS v1.1 sequence analysis software (Applied Biosystems).

### Tumor cell cultures and *in vitro* killing assays

All tumor cell lines were cultured in complete 10% RPMI 1640 (as above), maintained at 1×10^5^ up to 2×10^6^ cells/mL by dilution and splitting 1∶3 every 3–4 days.

For cytotoxicity assays, magnetically purified γδ PBL were pre-activated for 72 hours with 1–10 µg/mL αCD3 mAb (OKT3) or 1–10 nM HMB-PP either in the absence or presence of IL-2 (100 U/mL). Tumor cell lines were stained with CellTracer Far Red DDAO-SE (1 µM) (Molecular Probes, Invitrogen) and each 3×10^4^ tumour cells were incubated with 3×10^5^ γδ T-cells in RPMI devoid of activating compounds, for 3 hours at 37°C and 5% CO2 on a round-bottom 96 well plate. Cells were then stained with Annexin V-FITC and analyzed by flow cytometry.

### Confocal microscopy

Cells were stained at 4°C with mouse anti-human TCR Vgamma9-PC5 (Beckman Coulter) primary antibody, and with anti-mouse Alexa Fluor 633 (Invitrogen, Molecular Probes) secondary antibody. Cells were then fixed with 4% Paraformaldeheyde for 15 minutes at 4°C. Nuclear DNA content was stained for with DAPI Fluoromount G (Southern Biotech). Immunofluorescence microscopy was performed with a LSM 510 META confocal microscope (Zeiss). Separate images were collected with a 63× objective for each fluorochrome and then overlaid to obtain a multicolor image.

### Bioluminescent imaging of transplanted leukemia development in SCID mice

10^7^ Molt-4 T-cell leukemia cells stably expressing firefly luciferase and GFP were injected i.v. in groups of 6 NOD/SCID mice per experiment, either in isolation or together with 5×10^7^ γδ PBL (>80% Vγ9^+^), previously expanded and activated *in vitro* with 1 nM HMB-PP for 12 days. Treated mice received boosts of 5×10^7^ γδ PBL i.v. on day 14 and 10,000 U IL-2 i.p. twice every week, whereas control mice received only IL-2. All mice were analyzed on a weekly basis by *in vivo* imaging (IVIS, Caliper Lifesciences) upon intra-peritoneal injection of luciferin. Photon signals were quantified with LivingImage software (Caliper Lifesciences). Mouse body weight was measured weekly, and animals suffering from wasting (loss of over 20% of initial body weight) were sacrificed.

### Statistical analysis

Statistical significance of differences between subpopulations was assessed using Student's t-test and is indicated when significant as *, p<0.05; **, p<0.01; ***, p<0.001.

## Supporting Information

Figure S1 Exogenous IL-2 expands HMB-PP-activated Vg9+ T-cells and up-regulates their Th1 cytokine profile. (A) Absolute numbers of Vg9+ cells in PBMC cultures stimulated with HMB-PP (1 nM) or OKT3 (1 ug/ml), supplemented or not with IL-2 (100 U/ml). Cells were analyzed by flow cytometry and light microscopy (Mossbauer chamber cell counts). (B) Cytokine bead array (CBA) analysis of supernatants of MACS-sorted gd PBL (of which 80–90% Vg9+) after 24 hours of stimulation with HMB-PP or anti-CD3 mAb (OKT3). Represented is the ratio between the cytokine amounts produced in the presence (100 U/ml) and in the absence of IL-2. (C) Real-time PCR quantification of t-bet mRNA expression in activated Vg9Vd2 T-cells, normalized with Beta2-microglobulin. Cells were pre-incubated for 6 hours with the activating compounds (or kept in RPMI as control). Significant differences refer to cells cultured in RPMI in the absence of IL-2 (n = 3, *p<0.05 and **p<0.01).
(0.07 MB TIF)

Figure S2 Akt phosphorylation in response to IPP versus HMB-PP stimulation of gd PBL. MACS-sorted gd PBL were activated with 10 uM IPP or 1 nM HMB-PP for the indicated times. Cell lysates were analyzed by SDS-PAGE and immunoblotted for Phospho-Akt (P-Akt) or Beta-Actin on nitrocellulose membranes. Densitometry for P-Akt bands was normalized with Beta-Actin loading controls. Data correspond to the induction of Akt phosphorylation above basal levels, i.e., after subtraction of the unstimulated control levels.
(0.05 MB TIF)

Figure S3 Heatmap of non-stimulated, HMB-PP-treated and anti-CD3 mAb (OKT3)-treated gd T-cells. The DNA microarray expression value for each gene is normalized across the samples; levels greater than the mean in a given sample are colored in red, and those below the mean are depicted in blue. Exp1-3 are triplicate independent microarray experiments. Note the striking similarity between HMB-PP-treated and anti-CD3-treated samples.
(0.34 MB TIF)
