# Supplementary figures and images for: Highly Active Microbial Phosphoantigen Induces Rapid yet Sustained MEK/Erk- and PI-3K/Akt-Mediated Signal Transduction in Anti-Tumor Human γδ T-Cells
Source: PLoS One. 2009 May 21;4(5):e5657. doi: 10.1371/journal.pone.0005657 (PMC2682580; doi:10.1371/journal.pone.0005657)

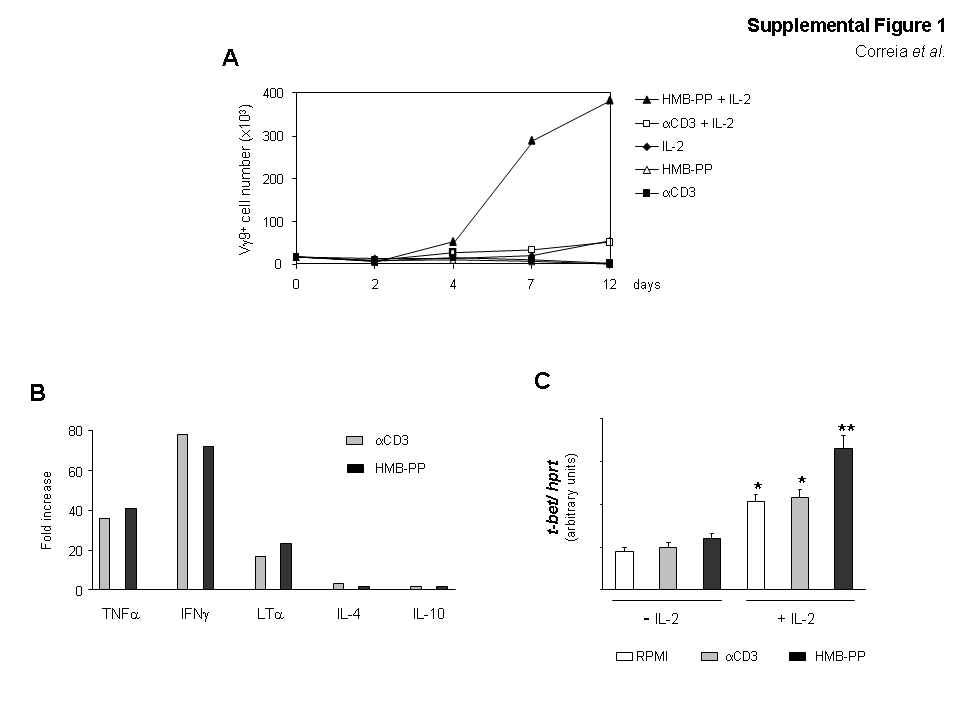

Supplement: Figure S1 — Exogenous IL-2 expands HMB-PP-activated Vg9+ T-cells and up-regulates their Th1 cytokine profile. (A) Absolute numbers of Vg9+ cells in PBMC cultures stimulated with HMB-PP (1 nM) or OKT3 (1 ug/ml), supplemented or not with IL-2 (100 U/ml). Cells were analyzed by flow cytometry and light microscopy (Mossbauer chamber cell counts). (B) Cytokine bead array (CBA) analysis of supernatants of MACS-sorted gd PBL (of which 80–90% Vg9+) after 24 hours of stimulation with HMB-PP or anti-CD3 mAb (OKT3). Represented is the ratio between the cytokine amounts produced in the presence (100 U/ml) and in the absence of IL-2. (C) Real-time PCR quantification of t-bet mRNA expression in activated Vg9Vd2 T-cells, normalized with Beta2-microglobulin. Cells were pre-incubated for 6 hours with the activating compounds (or kept in RPMI as control). Significant differences refer to cells cultured in RPMI in the absence of IL-2 (n = 3, *p<0.05 and **p<0.01). (0.07 MB TIF) [file pone.0005657.s001.tif]

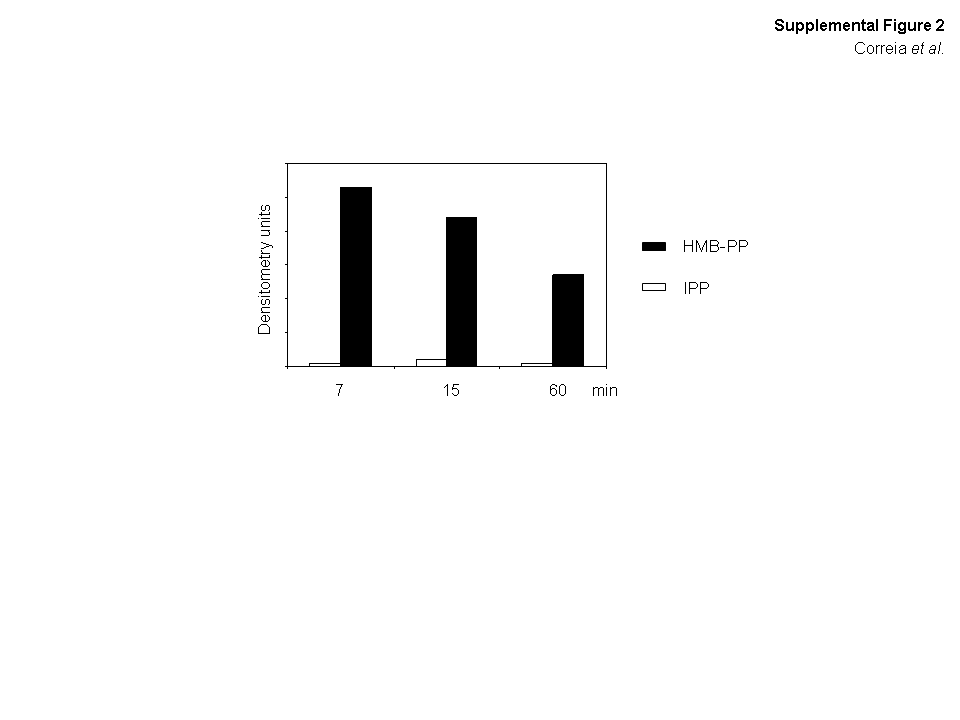

Supplement: Figure S2 — Akt phosphorylation in response to IPP versus HMB-PP stimulation of gd PBL. MACS-sorted gd PBL were activated with 10 uM IPP or 1 nM HMB-PP for the indicated times. Cell lysates were analyzed by SDS-PAGE and immunoblotted for Phospho-Akt (P-Akt) or Beta-Actin on nitrocellulose membranes. Densitometry for P-Akt bands was normalized with Beta-Actin loading controls. Data correspond to the induction of Akt phosphorylation above basal levels, i.e., after subtraction of the unstimulated control levels. (0.05 MB TIF) [file pone.0005657.s002.tif]

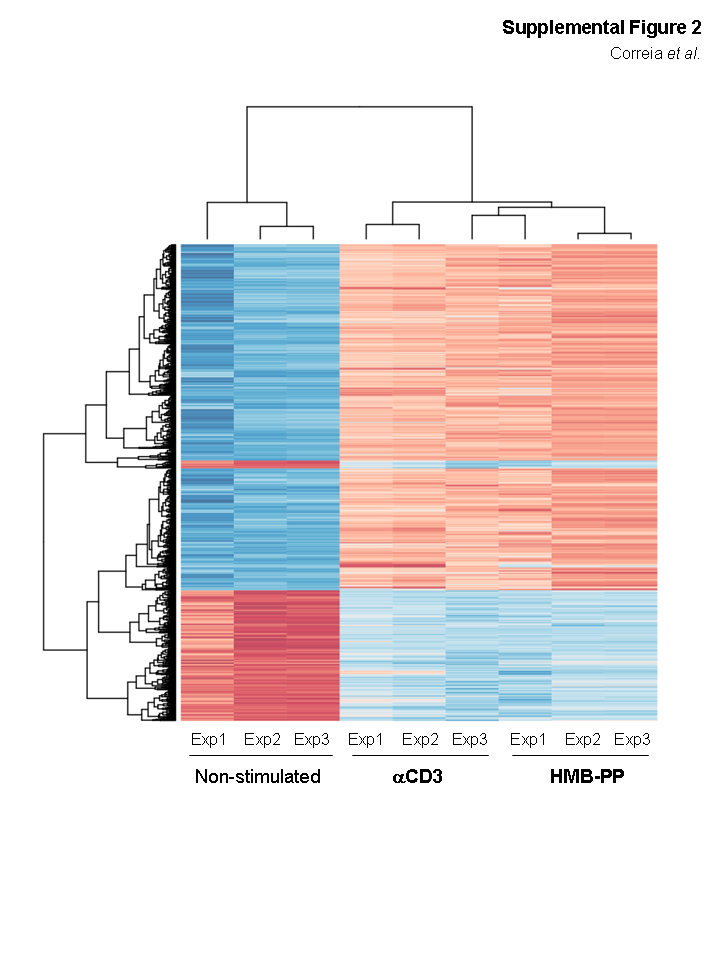

Supplement: Figure S3 — Heatmap of non-stimulated, HMB-PP-treated and anti-CD3 mAb (OKT3)-treated gd T-cells. The DNA microarray expression value for each gene is normalized across the samples; levels greater than the mean in a given sample are colored in red, and those below the mean are depicted in blue. Exp1-3 are triplicate independent microarray experiments. Note the striking similarity between HMB-PP-treated and anti-CD3-treated samples. (0.34 MB TIF) [file pone.0005657.s003.tif]
